# Access to Aboriginal Community-Controlled Primary Health Organizations Can Explain Some of the Higher Pap Test Participation Among Aboriginal and Torres Strait Islander Women in North Queensland, Australia

**DOI:** 10.3389/fonc.2021.725145

**Published:** 2021-07-28

**Authors:** Paramita Dasgupta, John R. Condon, Lisa J. Whop, Joanne F. Aitken, Gail Garvey, Mark Wenitong, Peter D. Baade

**Affiliations:** ^1^Cancer Council Queensland, Brisbane, QLD, Australia; ^2^Menzies School of Health Research, Charles Darwin University, Darwin, NT, Australia; ^3^National Centre for Epidemiology and Population Health, Research School of Population Health, Australian National University, Acton, ACT, Australia; ^4^School of Public Health, The University of Queensland, Brisbane, QLD, Australia; ^5^School of Public Health and Social Work, Queensland University of Technology, Brisbane, QLD, Australia; ^6^Institute for Resilient Regions, University of Southern Queensland, Brisbane, QLD, Australia; ^7^School of Exercise and Nutrition Science, Queensland University of Technology, Brisbane, QLD, Australia; ^8^Menzies Health Institute Queensland, Griffith University, Southport, QLD, Australia; ^9^School of Mathematical Sciences, Queensland University of Technology, Gardens Point, Brisbane, QLD, Australia

**Keywords:** cervical cancer, Pap test, Aboriginal and Torres Strait Islander, inequalities, Australia

## Abstract

**Background:**

Aboriginal and Torres Strait Islander Community-Controlled Health Organisations (ACCHOs) provides culturally appropriate primary care for Aboriginal and Torres Strait Islander people in Australia. The population of North Queensland has a higher proportion of Aboriginal and Torres Strait Islander people, a greater population coverage of ACCHOs, and higher cervical screening participation than the Rest of Queensland. The association between regional differences in the use of ACCHOs for cervical screening and variations in screening participation among Aboriginal and Torres Strait Islander women is currently unknown.

**Methods:**

This is a population-based study of 1,107,233 women, aged 20–69 years who underwent cervical screening between 2013 and 2017. Of these women, 132,972 (12%) were from North Queensland, of which 9% were identified as Aboriginal and Torres Strait Islander women (2% Rest of Queensland) through linkage to hospital records. Regional differentials in screening by Aboriginal and Torres Strait Islander status were quantified using participation rate ratios (PRRs) with 95% confidence intervals (CIs) from negative binomial regression models. Logistic regression was used to identify factors associated with Aboriginal and Torres Strait Islander women being screened at ACCHOs.

**Results:**

Aboriginal and Torres Strait Islander women from North Queensland (*versus*) Rest of Queensland had higher odds of screening at ACCHOs after adjusting for age and area-level variables. After adjustment for non-ACCHO variables, the regional differential in screening among Aboriginal and Torres Strait Islander women was significantly higher (PRR 1.28, 95% CI 1.20–1.37) than that among other Australian women [PRR = 1.11 (1.02–1.18)], but was attenuated on further adjustment for ACCHO variables, [PRR = 1.15, (1.03–1.28)] to become similar to the corresponding point estimate for other Australian women [PRR = 1.09, (1.01–1.20)]. However, the significant interaction between Aboriginal and Torres Strait Islander status and region (*p* < 0.001) remained, possibly reflecting the large cohort size. Screening participation increased with better access to health services for all women.

**Conclusions:**

Improving access to primary health care for Aboriginal and Torres Strait Islander women, especially through ACCHOs, may reduce existing disparities in cervical screening participation. Further gains will require greater levels of local community engagement and understanding of the experiences of screened Aboriginal and Torres Strait Islander women to inform effective interventions.

## Introduction

Australian Aboriginal and Torres Strait Islander women experience a disproportionately high burden of cervical cancer (1, 2) despite a national population-based cervical screening program (NCSP) (3, 4) which, combined with high uptake of human papillomavirus vaccine (5), has led to population cervical cancer incidence and mortality rates in Australia being among the lowest worldwide (6).

On December 1, 2017, a renewed NCSP was implemented with five-yearly primary human papillomavirus (HPV)-based screening for women aged 25–74, replacing the original two-year Papanicolaou (Pap) test for those aged 20–69 years (4). Although both pathways involve clinical collection of a cervical sample suggesting that some of the factors impacting screening participation may be similar, this remains untested as the first population-based participation data for the renewed program will not be available until after 2022 (4).

National data on participation of Aboriginal and Torres Strait Islander women in the previous (Pap test based) NCSP are unavailable because pathology report forms, the primary source of information for cervical screening registers, did not routinely record Aboriginal and Torres Strait Islander status (1, 7). However, state-based studies using record linkage to identify Aboriginal and Torres Strait Islander women have reported substantially lower participation for Aboriginal and Torres Strait Islander women that have persisted over at least 10 years in Queensland, Australia (8–10). These studies also found that participation was higher among Aboriginal and Torres Strait Islander women, and other Australian women, living in North Queensland compared to the rest of the state (8, 9). North Queensland in this context refers to the northernmost and north west region of Queensland, including the cities of Cairns and Townsville, and has a distinctive regional character and identity (11). For example, in North Queensland, a higher proportion of the population are from more remote or disadvantaged areas, a lower proportion are from affluent areas, and a higher proportion identified as Aboriginal and Torres Strait Australians compared to total Queensland.

Compared to the Rest of Queensland, North Queensland has a higher proportion of the population who are identified as Aboriginal and/or Torres Strait Islander people (12) or who live in regional, remote, or disadvantaged areas (13). It also has a higher population coverage (14, 15) of Australian government-funded Aboriginal and Torres Strait Islander Community-Controlled Health Organisations (ACCHOs) (16). Ratios of ACCHO locations to populations are higher in north and western parts of Queensland and lower in the eastern parts of the state, particularly so for the south-east corner of the state (14). ACCHOs provide comprehensive, culturally appropriate, and accessible primary health care specifically for Aboriginal and Torres Strait Islander people (17, 18). They aim to be responsive to the needs of the local community and enable Aboriginal and Torres Strait Islander people’s self-determination and empowerment (16, 18).

In Australia, cervical screening through the NCSP occurs mainly in primary care (with additional mobile health units) (1) and is provided at no cost for eligible women, though providers may charge a small service fee (19). Aboriginal and Torres Strait Islander women may either access mainstream or Aboriginal and Torres Strait Islander-specific primary health care services (mainly ACCHOs) (17). There is evidence that these services play a crucial role in delivering cervical screening (20, 21); in a semi-national clinical audit from 2005 to 2014, at least half of Aboriginal and Torres Strait Islander women who regularly attended these services had a two-yearly Pap test (21).

Previous studies have suggested that sustained participation in a program of continuous service improvement designed to identify and address barriers and facilitators to Pap smear screening led to higher cervical screening coverage among Aboriginal and Torres Strait Islander women at ACCHOs (21, 22). Successful strategies included targeted culturally relevant health education, local community involvement, and establishment of specific women’s health clinics with female practitioners (21, 22). Other facilitators include access to female practitioners and trained Aboriginal and Torres Strait Islander health workers to promote and perform screening tests which are associated with higher cervical (and breast) screening participation among screen-eligible Aboriginal and Torres Strait Islander women (23, 24). While enacting these enablers is important for long-term improvements in cervical screening, a more comprehensive understanding of the service and system level barrios (and facilitators) to screening is essential for the development of effective and culturally sensitive interventions to reduce the existing gap in cervical cancer incidence and mortality.

This population-based study used a large, linked dataset containing details of the cervical screening history and Aboriginal and Torres Strait Islander status of individual women to explore factors associated with attendance at ACCHOs for Pap tests among Aboriginal and Torres Strait Islander women living in North Queensland compared to the Rest of Queensland. We also quantified whether access to ACCHOs is associated with regional variations in five-year cervical screening participation among Aboriginal and Torres Strait Islander women and whether access to primary health care is associated with cervical screening among other Australian women.

## Methods

Ethical approval was obtained from the Queensland Metro South Human Research Ethics Committee (HREC/2018/QMS/44576). Data access and record linkage were approved by the office of the Director-General of Queensland Health, relevant data custodians, and the Queensland Data Linkage unit.

### Regions

The geographical unit was the 2016 Statistical Area Level 2 (SA2) from the 2016 Australian Statistical Geography Standard, defined by the Australian Bureau of Statistics (ABS) as representing a community that interacts together socially and economically (25).

The North Queensland region was defined approximately as that part of Queensland north of latitude −20°. This region covers more than a third of the total land area (39% or 680,000 km^2^) of Queensland (**Table 1**) and includes the major population centers of Cairns, Cooktown, Townsville, Mount Isa, and Charters Towers (**Supplementary File 1 Figure S1.1**). Based on 2016 SA2 boundaries, there are 80 SA2s that covered the entire North Queensland region without gaps or overlaps, each with varying land areas [median area 56 km^2^, interquartile range (IQR): 9 to 945 km^2^] and population (median: 5,924, IQR: 4,082 to 8,732).

**Table 1:** Regional characteristics, Queensland, Australia 2017.

Region characterisitcs^1-7^		
	North Queensland	Rest of Queensland
**Percent of geographical area covered**	39.3	60.7
**Percent of total Queensland population (persons)**	11.4	88.6
**Percent of population (persons) who…**		
are Aboriginal and Torres Strait Islander	14.4	3.3
live in remote areas	11.4	0.9
live in major cities	0.0	72.2
live in disadvantaged areas	26.4	17.1
live in affluent areas	10.1	19.8
live within 30 min of a Pap test provider	96.1	98.7
live within 30 min of an ACCHO Pap test provider	85.0	80.8
live more than 1 h of an ACCHO Pap test provider	5.9	2.8
live within 30 min of a non-ACCHO Pap test provider	94.9	98.7
live more than 1 h of a non-ACCHO Pap test provider	1.6	0.04
**Number of 2016 Statistical Area Level 2**	80	448

ACCHO, Aboriginal Community-Controlled Health Organisation.

1. Census and population data were obtained from the Australian Bureau of Statistics.

2. Indigenous population data for Queensland was obtained from the Queensland Treasury.

3. Statistical Area Level 2 (SA2) from the 2016 Australian Statistical Geography Standard (ASGS).

4. Remote areas were defined by the Remoteness Areas 2016 classification (combines Remote and Very Remote).

5. ‘Affluent areas’ are the 20% of most advantaged Statistical Areas 2 (SA2s) and ‘Disadvantaged areas’ are the 20% of most disadvantaged SA2s as defined by the 2016 SEIFA Index of Relative Socioeconomic Advantage and Disadvantage obtained from the Australian Bureau of Statistics.

6. Pap test providers based on medical centers or general practitioner practices. One center or practice may have multiple health professionals who provide Pap tests.

7. Based on travel time from a SA2 (2016) at screening to geocoded residential street address of a Pap test provider.

The remaining area of Queensland, comprising 448 SA2s, (median area 12 km^2^ IQR: 5 to 64 km^2^; population median 8,191; IQR: 5,289 to 12,074) is referred to here as “Rest of Queensland”.

### Study Cohort

The study cohort comprised all female residents of Queensland, aged 20 to 69 years, who had a Pap test between January 1, 2013 and December 31, 2017. Aboriginal and Torres Strait Islander women in the NCSP were identified through linking the population-based Queensland Health Pap Smear Register (PSR), which during the study period collated all Pap tests performed state-wide (with the renewed NCSP there has been a transition to the National Cancer Screening Register), and the Queensland Hospital Admitted Patient Data Collection (QHAPDC) (26) that has accurate information on Aboriginal and Torres Strait Islander status (27).

Full details of the data extraction and record linkage process have been described previously (8). Briefly, records were extracted from the Queensland Health PSR for all Pap tests (and cervical-related histology tests) performed between January 1, 2012 and December 31, 2017 for women aged 15–69 years, who had not opted off the register. Records for all women who had been discharged at least once from public and private Queensland hospitals between April 1, 2000 (July 1, 2007 for private hospitals) and December 31, 2017 (inclusive) were extracted from the QHAPDC for all women aged 15–69 years during the 2012–2017 calendar period. The two extracts were then linked using a combination of deterministic and probabilistic methods, including clerical review (26). Based on unpublished advice from the Linkage Unit, 81% of the Queensland Health PSR cohort was successfully linked to the QHAPDC.

Aboriginal and Torres Strait Islander status was determined using a standard majority-based algorithm (28, 29) with a woman in the Queensland Health PSR coded as Aboriginal and Torres Strait Islander if at least 50% of her linked QHAPDC records within the study period identified her as being Aboriginal and Torres Strait Islander. The ‘majority-based algorithm’ is one of four standard algorithms recommended by the Australian Institute of Health and Welfare (Australia’s national agency for health and welfare related statistics) for the assignment of Indigenous status, thereby ensuring consistency both within and across administrative data sets (28, 29). All other linked women, and those who did not match to at least one QHAPDC record, were classified as other Australian. Information on the ethnicity of other Australian women was not available.

Previous sensitivity analyses (28, 29) indicated that the proportion of Aboriginal and Torres Strait Islander women in the linked dataset ranged from 2.3 to 2.5% based on four standard algorithms.

### Geographical Area at Screening

Residential suburb and postcode for each Pap test record were mapped to the 2016 SA2 boundaries using population weighted geographic correspondence (30). If the address information for a given record was insufficient to assign the SA2, information from the closest (by date) record for the same woman with viable information was used. Women lacking geographical information for all records were excluded (n = 6,076, 79 Aboriginal and Torres Strait Islander).

Area-level socio-economic status (SES) was assessed using the 2016 census-based Index of Relative Socioeconomic Advantage and Disadvantage (IRSAD) from the Australian Bureau of Statistics (31). The IRSAD is a composite measure of SES incorporating multiple measures of advantage and disadvantage including occupation, income, and education (31). Each SA2 was allocated an IRSAD score and then ranked into five quintiles of increasing disadvantage (**Table 2**). Remoteness was measured using the 2016 Remoteness Areas (32) classification, a purely geographic measure of relative access to services. The five remoteness areas were major cities, inner regional, outer regional, remote, and very remote. Each SA2 was categorized as having a low (<2.0%) or high (≥2.0%) proportion of Aboriginal and Torres Strait Islander women based on the 2016 Australian Census (33); 2.0% was chosen as the cut-off because this allocated approximately half the Aboriginal and Torres Strait Islander female population to each category.

**Table 2:** Characteristics of screened women, Queensland 2013–2017 by region and Aboriginal and Torres Strait Islander status at time of first Pap test.

Variable	North Queensland (n = 132,972)		Rest of Queensland (n = 974,261)	
	Aboriginal and Torres Strait Islander (%) (n = 11,944)	other Australian (%) (n = 121,028)	Aboriginal and Torres Strait Islander (%) (n = 15,225)	other Australian (%) (n = 959,036)
**Age group (years)**				
20–29	33.8	24.3	36.6	23.4
30–39	26.3	23.9	24.7	24.4
40–49	21.3	22.6	20.5	22.6
50–59	12.5	18.0	12.4	17.5
60–69	6.1	11.2	5.8	12.1
**Aboriginal and Torres Strait Islander female (%)^1^**				
Low (<2.0%)	0.6	4.1	26.3	56.6
High (≥2.0%)	99.4	95.9	73.7	43.4
**Area-level disadvantage^2^**				
Most advantaged	0.7	4.8	8.0	23.5
Advantaged	5.5	20.1	14.4	24.2
Middle SES	14.5	23.5	17.1	20.7
Disadvantaged	25.1	27.8	24.7	16.0
Most disadvantaged	54.2	23.8	35.8	15.6
**Remoteness^3^**				
Major cities	0	0	52.8	74.3
Inner regional	0	0	32.9	20.6
Outer regional	59.6	92.3	10.7	4.2
Remote	18.2	5.9	1.5	0.5
Very remote	22.2	1.8	2.1	0.4
**Actual Pap test provider^4,5^**				
ACCHO	24.9	0.8	16.9	0.3
Non-ACCHO	60.2	90.9	72.2	93.3
Unknown	14.9	8.3	10.9	6.4
**Closest Pap test provider^4,6^**				
ACCHO	72.4	86.8	78.5	82.3
Non-ACCHO	18.0	1.5	0	0
Both	9.6	11.7	21.5	17.7
**Number ACCHO providers^4,7^**				
None	43.5	77.4	74.8	88.8
One	10.8	8.3	15.2	7.7
Two to four	23.1	11.6	9.2	3.3
Five or more	22.6	2.7	0.8	0.2
**Number non-ACCHO providers^4,7^**				
None	12.1	6.6	2.3	2.2
One	9.1	7.8	5.5	5.7
Two to four	29.7	32.5	22.1	22.1
Five to nine	30.5	27.5	31.5	37.5
10 or more	18.6	25.6	38.6	32.5
**Travel time closest ACCHO provider^6^**				
<30 min	80.9	86.4	76.8	81.7
30 min-1 hour	15.4	7.7	18.0	15.2
1-2 hours	3.4	5.3	3.9	2.9
2-5 hours	0.3	0.6	1.3	0.2
**Travel time closest non-ACCHO provider^6^**				
<30 min	81.2	96.9	95.9	98.4
30 min-1 hour	9.6	1.7	3.6	1.5
1-2 hours	1.4	0.1	0.5	0.1
2-5 hours	7.8	1.3	0	0

ACCHO, Aboriginal Community-Controlled Health Organisation; SES, socio-economic status.

1. Based on 2016 Australian Census.

2. Area-level disadvantage was defined by the 2016 SEIFA Index of Relative Socioeconomic Advantage and Disadvantage.

3. Remote areas were defined by the Remoteness Areas 2016 classification.

4. Provider refers to Pap test providers and are based on medical centers or general practitioner practices, that may have multiple health professionals who provide Pap tests.

5. Actual Pap test provider is the provider where a screened woman in the cohort had her first (index) Pap test during study period.

6. Based on travel distance from 2016 Australian Statistical Geography Statistical Area Level 2 (SA2) at screening to geocoded street address of a Pap test provider.

7. Number providers by 2016 Australian Statistical Geography Statistical Area Level 2 (SA2) at screening which is based on suburb and postcode of residence of a woman when screened.

### Pap Test Providers

The term ‘Pap test provider’ is used in this paper to refer to the health care center where a Pap test was performed. A center may have multiple medical professionals who carried out Pap tests.

Information on the name and street address of Pap test providers was extracted from the Queensland Health PSR records for all women in the study cohort and geocoded to assign the corresponding SA2. Each unique provider (based on name and address details) was classified as an ACCHO if it matched with the published names and address details of an ACCHO in Queensland sourced from the National Aboriginal Community Controlled Health Organisation website (34) and links to the individual websites of all National Aboriginal Community Controlled Health Organisation members that were accessed from there. Unmatched geocoded providers were classified as non-ACCHO. Providers with missing address information and those located interstate, which could not be geocoded, were categorized as ‘unknown’. Syntax searching in Stata was used during the matching process to consider the possibility of alternative spellings, synonyms, and abbreviations in the recorded provider information including Indigenous Health, IPHC, ACCHO, Aboriginal and Torres Strait Islander, Aboriginal & Torres Strait Islander Health, Aboriginal Health and ATSICHS, with this process refined through several iterations.

Geographical Information System software and a street network database were used to calculate road travel times from the centroid of residential SA2 of each woman at the time of screening to the geocoded location of the actual provider used for their Pap test. Corresponding travel times were also calculated from each provider to each of the 528 SA2s in Queensland to determine, for each SA2, the closest ACCHO or non-ACCHO facility. If both ACCHO and non-ACCHO health facilities were equidistant to a given SA2, the closest provider was set to ACCHO.

### Estimated Resident Population

Population estimates for Queensland women by Aboriginal and Torres Strait Islander status, age, and SA2 across Queensland over the study period (35) were adjusted using age-specific hysterectomy fractions (1) to exclude women who had a hysterectomy from the population eligible for cervical screening. We used the same fraction for all women across all geographical areas because these fractions are not available by SA2 or by Aboriginal and Torres Strait Islander status (9). Although lower hysterectomy rates for Aboriginal and Torres Strait Islander women have been reported (36), the impact of this for our cohort is likely to be minimal given the younger age distribution for screened Aboriginal and Torres Strait Islander women (**Table 2**) and the lower hysterectomy rates among younger women (1).

We had no data on the catchment population for each of the providers, hence screening participation rates could not be calculated based on ‘actual Pap test provider’.

### Statistical Analysis

All statistical analyses were performed using Stata/SE (Version 16.1, Special Edition; Stata Corporation, College Station, TX, United States). Maps were generated using MapInfo Professional (version 16.0, Pitney Bowes, Stamford CT, United States).

#### Screening Participation

The outcome variable for this analysis was the cervical screening participation rate within the five-year time period of 2013 to 2017. Overall participation for the five-year screening interval of 2013–2017 was calculated as the age-specific number of screened women aged 20–69 divided by the estimated resident population [ERP, (**Supplementary File 1 Figure S1.2**), directly age-standardized (2001 Australian standard population). Estimates were calculated separately for Aboriginal and Torres Strait Islander women and other Australian women over the two regions: North Queensland and Rest of Queensland. Five-year intervals were chosen to be consistent with the current five-year screening interval for the NCSP (4). The participation rate measure is person-based, with a woman considered to have participated within the 2013–2017 time period if she was screened at least once during that time period. Women who had multiple screens within that 5-year time period were only counted once.

#### Regional Differential in Screening by Aboriginal and Torres Strait Islander Status

The regional differential (North Queensland *versus* Rest of Queensland) in five-year screening participation was quantified using negative binomial regression models, stratified by Aboriginal and Torres Strait Islander status. These models were chosen to account for extra-Poisson variation in the data. Details of the first (index) Pap test in the five-year study period were retained. The outcome variable was the number of screened women with the exposure variable being the corresponding population defined by age group and SA2.

Two models were fitted: the first was adjusted for region, age at screening, area-level SES, remoteness, and Aboriginal and Torres Strait Islander population (%). We further adjusted for ACCHO variables: closest Pap test provider type (ACCHO, non-ACCHO) and number of ACCHO providers (per screening SA2) as well as number of non-ACCHO providers (per screening SA2). Variables relating to travel time to closest ACCHO or non-ACCHO Pap test provider were not included in the final multivariate model because their inclusion did not improve model fit (*i.e.*, *p* ≥ 0.20 for likelihood ratio tests of models with and without each variable). Their inclusion or exclusion also did not alter the magnitude and confidence intervals of the coefficients for the other key variables in the models.

All models were initially stratified by Aboriginal and Torres Strait Islander status, and then a combined fully adjusted model (non-ACCHO and ACCHO variables) including interaction terms between each variable and Aboriginal and Torres Strait Islander status was fitted to test whether individual effects were different for Aboriginal and Torres Strait Islander than other Australian women.

Results are presented as Participation Rate Ratios (PRRs) with 95% Confidence intervals (CIs) which were calculated by exponentiating the coefficients from the negative binomial models. Individual coefficients and interaction terms were assessed with the Wald test (significant if *p* ≤ 0.05, two-sided).

#### Predictors of Screening at ACCHOs for Aboriginal and Torres Strait Islander Women

The study cohort for this separate analysis was restricted to Aboriginal and Torres Strait Islander women for whom there was sufficient locational information to classify the provider of their index Pap test as ACCHO or non-ACCHO. Logistic regression was then used to identify independent factors associated with Aboriginal and Torres Strait Islander women being screened at an ACCHO.

Models were developed in a stepwise manner, starting with the full model that included age, region, SES, remoteness, and Aboriginal and Torres Strait Islander female population (%). Variables were then dropped from subsequent models based on likelihood ratio tests (p ≥ 0.20). Once dropped, each variable was given the opportunity to re-enter subsequent models.

Second-order interaction terms between each variable in the final main-effects multivariable model and region were also fitted to test whether effects varied by region.

Exponentiated coefficients from these models are presented as odds ratios (ORs) with 95% CI.

## Results

### Regional Characteristics

In 2017, about 11.4% of the total population of Queensland lived in North Queensland. A higher proportion of the North Queensland population (than the Rest of Queensland) were Aboriginal and Torres Strait Islander people (14.4 *versus* 3.3%) and lived in remote (11.4 *versus* 0.9%) or disadvantaged (26.4 *versus* 17.1%) areas (**Table 1**). North Queensland also had a higher coverage of ACCHOs by population of Aboriginal and Torres Strait Islander women aged 20–69 years (**Supplementary File 1 Figure S1.3** **)**. However, 10% of SA2s in North Queensland were at least 1 h travel time from their closest ACCHO compared to around 4% in Rest of Queensland.

### Study Cohort

The initial cohort comprised 1,169,762 (28,530 Aboriginal and Torres Strait Islander) female residents of Queensland aged 20–69 years with known geographical information on address at screening who underwent a total of 2,396,250 Pap tests between January 1, 2012 and December 2017. For consistency with five-year screening rates, women who were screened in 2012 were dropped to give the final cohort of 1,107,233 women (27,169 Aboriginal and Torres Strait Islander) with 1,992,810 Pap test records.

Of these women, 132,972 (12.0% of cohort) lived in North Queensland with 233,019 Pap test records, of which 11,944 (8.9%) were identified as Aboriginal and Torres Strait Islander with 20,102 records. There were 974,261 women who lived in the Rest of Queensland, of which 15,225 (1.6%) were Aboriginal and Torres Strait Islander with 25,681 records (**Table 2**).

The proportion of Aboriginal and Torres Strait Islander women who had their index screen at an ACCHO was higher in North Queensland than the Rest of Queensland; with <1% of other Australian women screened at an ACCHO (**Table 2**). Most women who screened at an ACCHO were Aboriginal and Torres Strait Islander (North Queensland 76%; Rest of Queensland 52%).

More than four out of five women (86% other Australian; 80% Aboriginal and Torres Strait Islander) had their index screen at their closest provider.

### Screening Participation

#### Overall

Overall, Aboriginal and Torres Strait Islander women had significantly lower participation than other Australian women for both regions during 2013–2017 (**Supplementary File 2**). However, participation was higher for Aboriginal and Torres Strait Islander women in North Queensland than the Rest of Queensland. For example, the five-year participation rate was higher in North Queensland than Rest of Queensland by around 30% for Aboriginal and Torres Strait Islander women and 8% for other Australian women.

#### Regional Differential in Screening by Aboriginal and Torres Strait Islander Status

After adjustment for age, area-level SES, remoteness, and percentage of the female population who were Aboriginal and Torres Strait Islanders, Aboriginal and Torres Strait Islander women living in North Queensland were 28% more likely (PRR 1.28, 95% CI 1.20–1.37) to have participated in cervical screening than those from the Rest of Queensland. This regional differential was significantly higher than that for other Australian women (PRR = 1.11, 95% CI 1.02–1.18) with a significant interaction between Aboriginal and Torres Strait Islander status and region.

**Figure 1:**
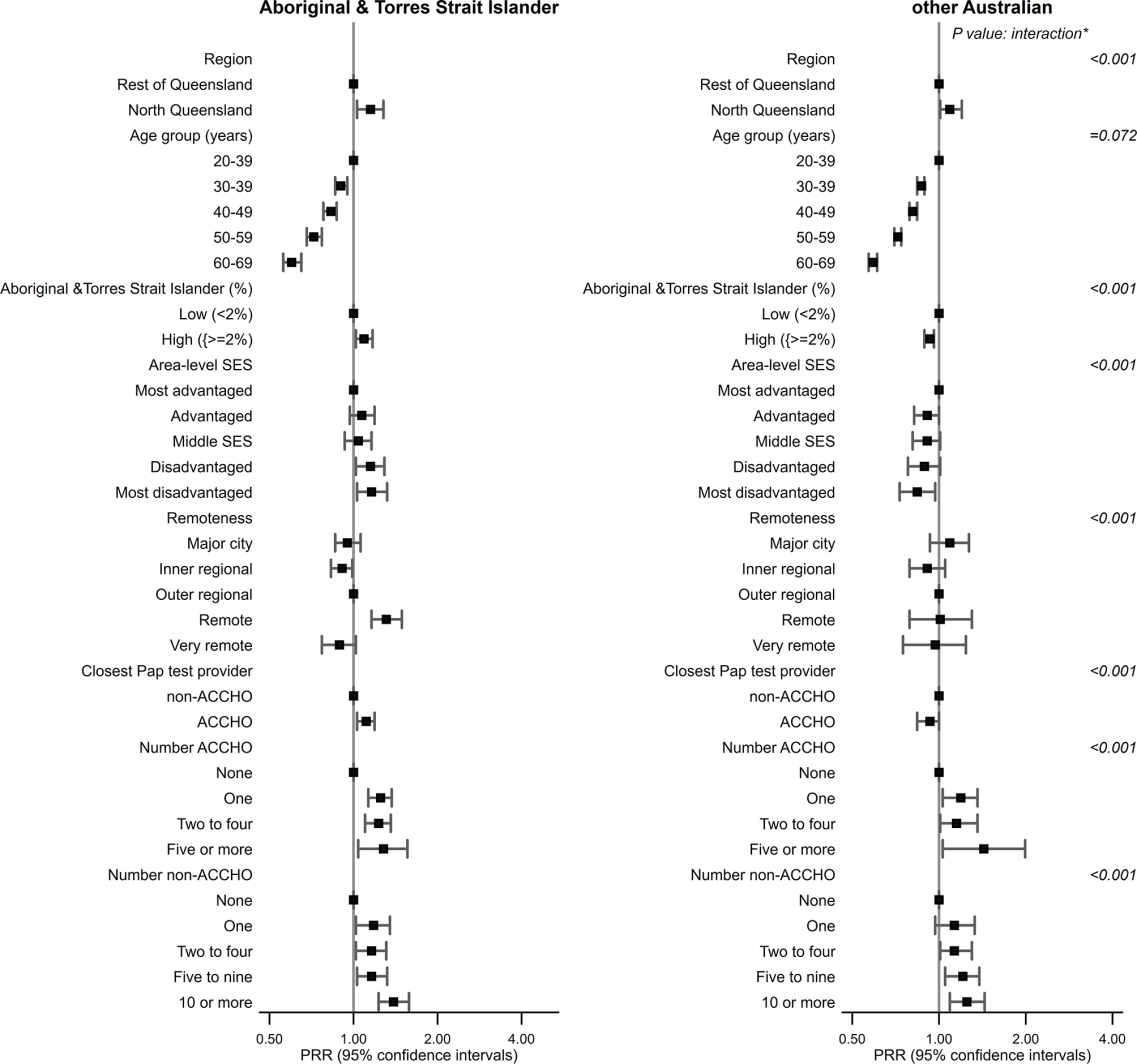
Plot of participation rate ratios (PRRs) with 95% confidence intervals for factors associated with screening participation, by region and Aboriginal and Torres Strait Islander status, Queensland (QLD) 2013–2017. SES, socio-economic status; ACCHO, Aboriginal Community-Controlled Health Organisation; PRR, Participation Rate Ratios. *interaction term between Aboriginal & Torres Strait Islamder status and each variable.

After further adjustment for closest Pap test provider type (ACCHO, non-ACCHO), number of ACCHO providers (per screening SA2), and number of non-ACCHO providers (per screening SA2), the regional differential for Aboriginal and Torres Strait Islander women was reduced to 15% (PRR = 1.15, 95% CI 1.03–1.28) (**Table 3** and **Figure 1**) similar to the corresponding point estimate for other Australian women. The significant interaction between Aboriginal and Torres Strait Islander status and region, however, remained, possibly reflecting the large cohort size.

**Table 3:** Participation rate ratios (PRR) [95% CI] for cervical screening by region, for Aboriginal and Torres Strait Islander women aged 20–69 years, Queensland, Australia, 2013–2017.

Variable	Adjusted Participation rate ratios [95% CI]^1,2,3^		Interaction (Aboriginal and Torres Strait Islander status, variable) *p-*value^4^
	Aboriginal and Torres Strait Islander	other Australian	
**Region**	*p* < 0.001	*p* = 0.011	*P* < 0.001
Rest of QLD	1.00	1.00	
North QLD	1.15 [1.03, 1.28]	1.09 [1.01, 1.20]	
**Age group (years)**	*p* < 0.001	*p* < 0.001	*P* = 0.072
20–29	1.00	1.00	
30–39	0.90 [0.86, 0.95]	0.87 [0.84, 0.89]	
40–49	0.83 [0.78, 0.87]	0.81 [0.79, 0.84]	
50–59	0.72 [0.68, 0.77]	0.72 [0.70, 0.74]	
60–69	0.60 [0.56, 0.65]	0.59 [0.57, 0.61]	
**Aboriginal and Torres Strait Islander female (%)^5^**	*p* < 0.001	*p* < 0.001	*p* < 0.001
Low (<2.0%)	1.00	1.00	
High (≥2.0%)	1.09 [1.02, 1.17]	0.93 [0.89, 0.96]	
**Area-level disadvantage^6^**	*p* = 0.033	*p* = 0.078	*p* < 0.001
Most advantaged	1.00	1.00	
Advantaged	1.07 [0.97, 1.19]	0.91 [0.82, 1.00]	
Middle SES	1.04 [0.93, 1.16]	0.91 [0.81, 1.01]	
Disadvantaged	1.15 [1.02, 1.29]	0.89 [0.78, 1.01]	
Most disadvantaged	1.16 [1.03, 1.32]	0.84 [0.73, 0.97]	
**Remoteness^7^**	*p* = 0.004	*p= 0.056*	*p* < 0.001
Major cities	0.95 [0.86, 1.06]	1.09 [0.93, 1.27]	
Inner regional	0.91 [0.83, 0.99]	0.91 [0.79, 1.05]	
Outer regional	1.00	1.00	
Remote	1.31 [1.16, 1.49]	1.01 [0.79, 1.30]	
Very remote	0.89 [0.77, 1.02]	0.97 [0.75, 1.24]	
**Closest Pap test provider^8,9,^**	*p* < 0.001	*p* = 0.062	*p* < 0.001
Non-ACCHO	1.00	1.00	
ACCHO^10^	1.11 [1.03, 1.19]	0.93 [0.84, 1.00]	
**Number ACCHO providers^8,11^**	*p* < 0.001	*p* = 0.019	*p* < 0.001
None	1.00	1.00	
One	1.25 [1.13, 1.37]	1.19 [1.03, 1.36]	
Two to four	1.23 [1.10, 1.36]	1.15 [1.01, 1.36]	
Five or more	1.28 [1.04, 1.56]	1.43 [1.03, 1.99]	
**Number non-ACCHO providers^8,11^**	*p* < 0.001	*p* = 0.013	*p* < 0.001
None	1.00	1.00	
One	1.18 [1.02, 1.37]	1.13 [0.97, 1.33]	
Two to four	1.16 [1.02, 1.31]	1.13 [1.01, 1.30]	
Five to nine	1.16 [1.03, 1.32]	1.21 [1.05, 1.38]	
10 or more	1.39 [1.23, 1.58]	1.25 [1.09, 1.44]	

ACCHO, Aboriginal Community-Controlled Health Organisation; CI, confidence Interval.

1. Estimated using negative binomial models, with outcome being number of screened women and offset the number of eligible women.

2. P-values from Wald’s joint test of coefficients for multivariate negative binomial regression.

3. Estimated from fully adjusted main effect negative binomial models stratified by Aboriginal and Torres Strait Islander status.

4. P-values from Wald’s X^2^ test for interaction effect from fully adjusted main-effects model with interaction term between each independent variable and Aboriginal and Torres Strait Islander status.

5. Based on 2016 Australian Census.

6. Area-level disadvantage was defined by the 2016 SEIFA Index of Relative Socioeconomic Advantage and Disadvantage.

7. Remote areas were defined by the Remoteness Areas 2016 classification.

8. Provider refers to provider of a Pap test and is based on medical centers or general practitioner practices that may have multiple health professionals who provide Pap tests.

9. Based on travel distance from 2016 (SA2) at screening to geocoded street address of a Pap test provider.

10. The category ACCHO includes those SA2’s for which the closest Pap test provider is either an ACCHO or both (ACCHO, non-ACCHO).

11. Number providers by 2016 Australian Statistical Geography Statistical Area Level 2 (SA2) for a woman at screening.

For both groups of women, screening participation was lower in older women. For Aboriginal and Torres Strait Islander women only, participation was higher among those from remote areas or areas with higher Aboriginal and Torres Strait Islander female population (**Figure 1**). While for Aboriginal and Torres Strait Islander women, screening was higher among more disadvantaged women, it decreased with increasing disadvantage for other Australian women. The effect of age did not vary by Aboriginal and Torres Strait Islander status (**Table 3**), whereas the corresponding interaction term for SES, remoteness, or Aboriginal and Torres Strait Islander female population (%) were statistically significant.

Aboriginal and Torres Strait Islander women were 11% more likely to be screened if the closest Pap test provider was an ACCHO than a non-ACCHO (**Table 3**). Screening participation increased with better access to any Pap test provider (ACCHO, non-ACCHO) as measured by the number of corresponding providers (per screening SA2) for both Aboriginal and Torres Strait Islander and other Australian women.

#### Predictors of Screening at ACCHOs for Aboriginal and Torres Strait Islander Women

The study cohort for this analysis consisted of 24,590 Aboriginal and Torres Strait Islander women, who had their index Pap test either at an ACCHO or non-ACCHO in Queensland. Of these women, 10,606 (43.1%) women lived in North Queensland and 13,984 (56.9%) in Rest of Queensland.

**Figure 2:**
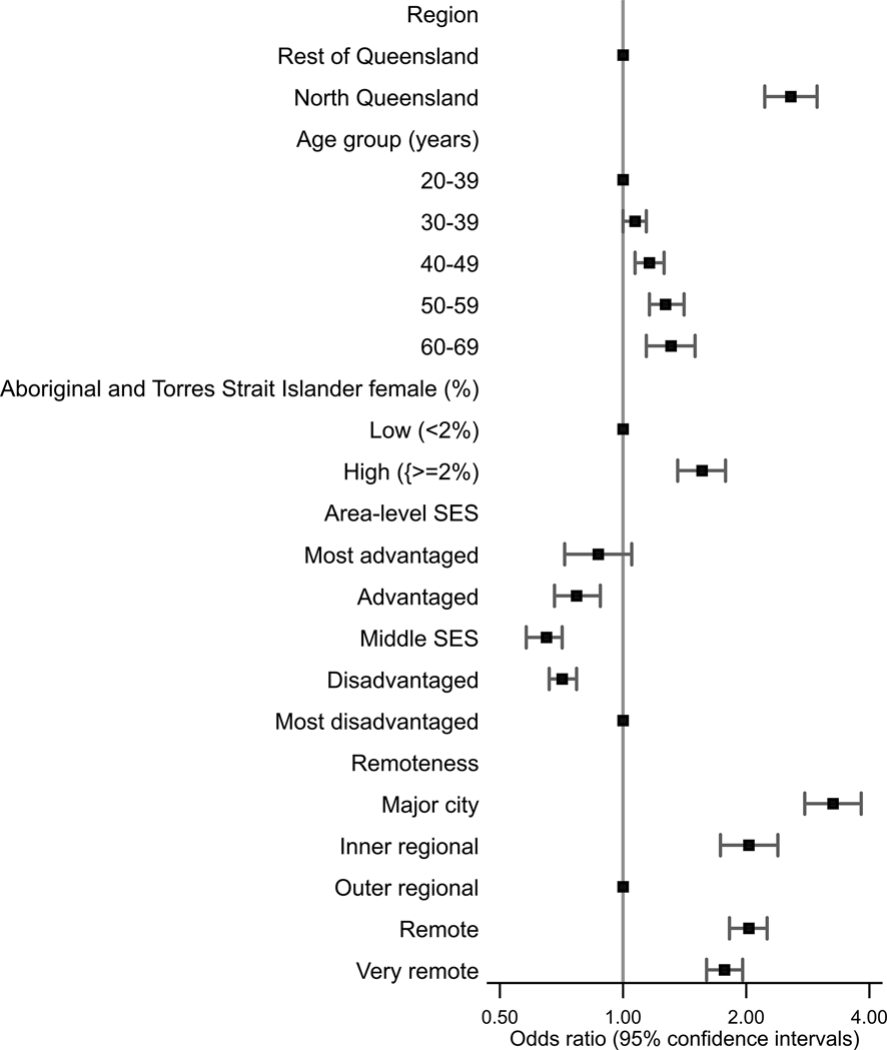
Plot of odds ratios with 95% confidence intervals for factors associated with Aboriginal and Torres Strait Islander women attending an Aboriginal community-controlled health organization (ACCHO) for a Pap test, Queensland, 2013–2017.

After adjusting for age and area-level variables, Aboriginal and Torres Strait Islander women from North Queensland (compared to Aboriginal and Torres Strait Islander women in the Rest of Queensland) were 2.6 times more likely to have their index screen at an ACCHO (**Table 4** and **Figure 2**). Use of ACCHOs for Pap tests was also independently higher among older women and those from areas with higher Aboriginal and Torres Strait Islander female population (%) or disadvantaged areas. Those not living in outer regional areas were also more likely to be screened at an ACCHO (**Table 4**).

**Table 4:** Participation rate ratios (PRRs) [95% CI] for cervical screening by region, for Aboriginal and Torres Strait Islander women aged 20–69 years, Queensland, Australia, 2013–2017.

	Adjusted Odds ratios ACCHO *versus* non-ACCHO (95% CI)^1,2,3^
	North Queensland
**Region**	*p* < 0.001
Rest of Queensland	1.00
North Queensland	2.57 [2.22, 2.98]
**Age group (years)**	*p* < 0.001
20–29	1.00
30–39	1.07 [1.00, 1.14]
40–49	1.16 [1.07, 1.26]
50–59	1.27 [1.16, 1.41]
60–69	1.31 [1.14, 1.50]
**Aboriginal and Torres Strait Islander female (%)^4^**	*p* < 0.001
Low (<2.0%)	1.00
High (≥2.0%)	1.56 [1.36, 1.78]
**Area-level disadvantage^5^**	*p* < 0.001
Most advantaged	0.87 [0.72, 1.05]
Advantaged	0.77 [0.68, 0.88]
Middle SES	0.65 [0.58, 0.71]
Disadvantaged	0.71 [0.66, 0.77]
Most disadvantaged	1.00
**Remoteness^6^**	*p* < 0.001
Major cities	3.26 [2.78, 3.82]
Inner regional	2.03 [1.73, 2.39]
Outer regional	1.00
Remote	2.03 [1.82, 2.25]
Very remote	1.77 [1.60, 1.96]

ACCHO, Aboriginal Community-Controlled Health Organisation; CI, confidence interval.

1. Estimated using fully adjusted main-effect logistic regression models.

2. P-values from Wald’s joint test of coefficients for multivariate logistic regression.

3. ACCHO Aboriginal Community-Controlled Health Organisation (ACCHOs) are community-controlled health services designed to meet the primary healthcare needs of Aboriginal and Torres Strait Islander people.

4. Based on 2016 Census.

5. Area-level disadvantage was defined by the 2016 SEIFA Index of Relative Socioeconomic Advantage and Disadvantage.

6. Remote areas were defined by the Remoteness Areas 2016 classification.

There was no statistical evidence that the effect of SES, the percent of the population who were Aboriginal and Torres Strait Islander or remoteness varied by region. However, the interaction between age group or SES and region was statistically significant. Increasing age was associated with higher odds of screening at an ACCHO only among Aboriginal and Torres Strait Islander women in Rest of Queensland, while those living in most disadvantaged areas were more likely to be screened at an ACCHO in North Queensland.

## Discussion

Cervical screening participation in 2013–2017 was higher among all women in North Queensland than among those in the Rest of Queensland. Although adjusting for ACCHO-related variables (closest Pap test provider type, number of ACCHO and non-ACCHO providers) reduced this regional difference among Aboriginal and Torres Strait Islander women, some regional variations remained, the magnitude of which was similar for Aboriginal and Torres Strait Islander and other Australian women. In other words, despite these adjustments, screening among Aboriginal and Torres Strait Islander women from North Queensland was still higher than the rest of the state. In addition, Aboriginal and Torres Strait Islander women from North Queensland were more likely to have their index Pap test at an ACCHO than those from Rest of Queensland even after adjustment for age and area-level factors.

This study indicates that access to ACCHOs may explain at least part of the regional variation in cervical screening participation for Aboriginal and Torres Strait Islander women in Queensland. There is evidence that physical access to ACCHOs and their coverage relative to Aboriginal and Torres Strait Islander population vary geographically across Australia (14, 15). In particular, there appear to be fewer ACCHOs (and other government-funded Aboriginal and Torres Strait-specific primary care organizations) in Central and Eastern Queensland, whereas higher population-based coverage of ACCHOs has been reported for North Queensland (14, 15).

The key goal of ACCHOs is to deliver comprehensive and culturally competent primary health care that is accessible and appropriate to the needs of Aboriginal and Torres Strait Islander people (17, 37). ACCHOs often employ Aboriginal and Torres Strait Islander health workers (37) and facilitate increased local community involvement, empowerment, and self-determination (16, 18).

Although there is a greater choice of screening providers in more urban areas, such services may not always be accessible or appropriate for Aboriginal and Torres Strait Islander women reflecting social, cultural, and system-level factors. Lower numbers of ACCHOs and other organizations providing primary health care specifically for Aboriginal and Torres Strait Islander people are also located in major cities compared to rural areas (17). By contrast, in remote and very remote areas, ACCHOs may be the only primary health care option for Aboriginal and Torres Strait Islander people (37), although only a very small proportion (4%) of other Australians are screened at an ACCHO. These factors may be reflected in the higher screening participation among Aboriginal and Torres Strait Islander women (but not other Australian women) from remote areas (*versus* major cities) in this study.

Moreover, while participation decreased with increasing disadvantage for other Australian women, consistent with overall higher cervical screening in affluent areas (4), the association was reversed for Aboriginal and Torres Strait Islander women. While we lack information to explore reasons for this differential in this study, the possible contribution of various initiatives for improving health of Aboriginal and Torres Strait Islander people from more disadvantaged areas (37, 38) should be explored in future studies.

While access to screening services for Aboriginal and Torres Strait Islander women through community driven and culturally informed health care organizations such as ACCHOs is likely to improve screening participation, other factors are also important. Although beyond the scope of this study, various geographical, organizational, and environmental factors have been previously associated with variations in cervical screening use across Aboriginal and Torres Strait Islander-specific primary health care services in Australia (21, 22). Moreover, a recent qualitative study designed to better understand the experiences of screened Aboriginal and Torres Strait Islander women suggested that in addition to personal factors (such as having control of their health), open discussion about screening and strong and trusting relationships with health professionals facilitated increased screening (39). This study also identified key logistical barriers to screening including competing demands, scheduling issues, and confidentiality concerns notably among rural health professionals. Proposed service-level strategies to improve screening participation included locally relevant community engagement, culturally tailored resources, flexible service provision, and better access to female (including Aboriginal and Torres Strait Islander) practitioners especially in rural areas (21, 22, 39). A greater understanding of systemic and local barriers (and enablers) impacting service delivery is crucial for ongoing innovations to maximize the role of ACCHOs in cervical screening for Aboriginal and Torres Strait Islander women. To be successful, any such initiative must be based on the perspectives and experiences of Aboriginal and Torres Strait Islander people as a core component.

It is likely that ACCHOs in remote areas employ more female health practitioners (16, 17) than urban areas. Greater availability of female especially Indigenous health practitioners and women’s only health clinics have also been identified as enablers of cervical screening among Aboriginal and Torres Strait Islander women (22, 23, 39). Given that all North Queensland is deemed to be either outer regional or remote with no urban areas, it is plausible that higher screening participation reflects better access to female practitioners.

After full adjustment for provider-related variables, the regional differential in screening was similar for Aboriginal and Torres Strait Islander and other Australian women. The factors impacting higher cervical screening participation that were evident for both groups of women in North Queensland are unknown. While improving access to screening providers may help reduce existing disparities, further research is required to understand other facilitators and/or barriers to cervical screening in Australia. These are likely to include patient, provider, logistical and health system factors (40, 41). It is also important to better understand how to best facilitate access to and the acceptability of self-collection of samples for HPV-based cervical testing, which is currently only available to women aged 30 years and over who are either never-screened or are overdue for screening (by at least two years) (4).

### Strengths and Limitations

Strengths of this study include the large population-based cohort with coverage until the end of the previous national cervical screening program in December 2017 and identification of screened Aboriginal and Torres Strait Islander women through record linkage to inpatient hospital records. Pap test providers were identified as ACCHOs based on publicly available information sourced from the National Aboriginal Community Controlled Health Organisation website (34) and individual homepages of member organizations listed therein. Given that the National Aboriginal Controlled Health Organisation is the peak leadership body for all ACCHOs in Australia (34) provides some confidence that our search efforts were representative of available knowledge. Locational information on Pap test providers enabled us to determine the closest provider (at health service level) for each SA2 in Queensland.

Limitations include issues related to data-linkage issues (8–10), Aboriginal and Torres Strait Islander identification (8–10), numerator–denominator bias in that they were both sourced from different datasets, geographical mapping based on self-reported suburb and postcode rather than a validated full street address, and the well-documented challenges of accurately estimating the population of Aboriginal and Torres Strait Islander people (42, 43), although we used the best available published small-area population estimates (35). Moreover while Aboriginal and Torres Strait Islander identification in the QHAPDC database is considered to be adequate for research purposes (27), it is inevitable that some women would have been misclassified due to errors in record linkage process or incomplete self-identification. We also lacked capacity to look at screening participation separately for Aboriginal *versus* Torres Strait Islander women.

Our measure of accessibility was based on area-level travel time to closest Pap test provider. Not all women in our cohort would have had their Pap test at their closest provider; however given the high correlation between actual and closest Pap test increases confidence in reported estimates. There was no data on the catchment population for each provider to enable estimation of the screening participation based on the ‘actual Pap test provider’.

This is an ecological analysis of a large population-based cohort of women who have participated in cervical screening, as such the SES measure used reflects the average level of disadvantage of the population living in each small area. These measures cannot be used to infer how individuals from the same area may differ from each other in their SES or how these differences are reflected in their screening behavior.

## Conclusions

The difference in cervical screening participation among Aboriginal and Torres Strait Islander women in North Queensland *versus* Rest of Queensland was reduced after adjusting for ACCHO-related factors suggesting that access to ACCHO may explain some of the regional differential. That participation was higher among all women from North Queensland in areas with more Pap test providers suggests that creating more opportunities for cervical screening especially in areas with currently poor access to primary health care may be warranted. Prioritizing the involvement, collaboration, and self-determination of Aboriginal and Torres Strait Islander people in all aspects of implementation and service delivery is crucial for equitable screening participation. Better understanding of the experiences of screened Aboriginal and Torres Strait Islander women is also important to inform tailored interventions that overcome both logistical and systemic barriers to screening.

Patterns of health care utilization among Aboriginal and Torres Strait Islander women in Australia are relevant not only to the Australian context but also for Indigenous and other disadvantaged populations around the world when considering the extent of disparities in their access to health services and the possible factors contributing to them.

## Data Availability Statement

The data analyzed in this study is subject to the following licenses/restrictions: Data analyzed for this paper are not able to be shared on any publicly available repository due to legal and confidentiality requirements. Requests to access these datasets should be directed to Health Innovation, Investment and Research Office; Department of Health, Queensland Government; https://www.health.qld.gov.au/hiiro.

## Ethics Statement

Ethical approval was obtained from the Queensland Metro South Human Research Ethics Committee (HREC/2018/QMS/44576). Written informed consent for participation was not required for this study in accordance with the national legislation and the institutional requirements.

## Author Contributions

The authors PB, PD, JC and JA devised the project and the main conceptual ideas, PB coordinated the study. PD carried out the statistical analysis. PD drafted the manuscript. PB contributed to the original draft of the manuscript and all authors. PB, PD, LW, JC, GG, MW, and JA refined and approved the submitted version. All authors contributed to the article and approved the submitted version.

## Funding

This work was made possible by the generous support of the Jack and Madeleine Little Foundation, and the E Robert Hayles & Alison L Hayles Charitable Trust. GG is supported by National Health and Medical Research Council (NHMRC) Investigator Grant (#1176651). LW is supported by a NHMRC Early Career Fellowship (#1142035). Funding bodies had no role in the study design, collection, analysis, and interpretation of data, writing of this article or the decision to submit this article for publication.

## Conflict of Interest

The authors declare that the research was conducted in the absence of any commercial or financial relationships that could be construed as a potential conflict of interest.

## Publisher’s Note

All claims expressed in this article are solely those of the authors and do not necessarily represent those of their affiliated organizations, or those of the publisher, the editors and the reviewers. Any product that may be evaluated in this article, or claim that may be made by its manufacturer, is not guaranteed or endorsed by the publisher.
