# Supplementary material for: Access to Aboriginal Community-Controlled Primary Health Organizations Can Explain Some of the Higher Pap Test Participation Among Aboriginal and Torres Strait Islander Women in North Queensland, Australia
Source: Front Oncol. 2021 Jul 28;11:725145. doi: 10.3389/fonc.2021.725145 (PMC8355598; doi:10.3389/fonc.2021.725145)

Supplementary file Additional figures

1 Figure S1.1 Map of Queensland by region: North Queensland and Rest of Queensland

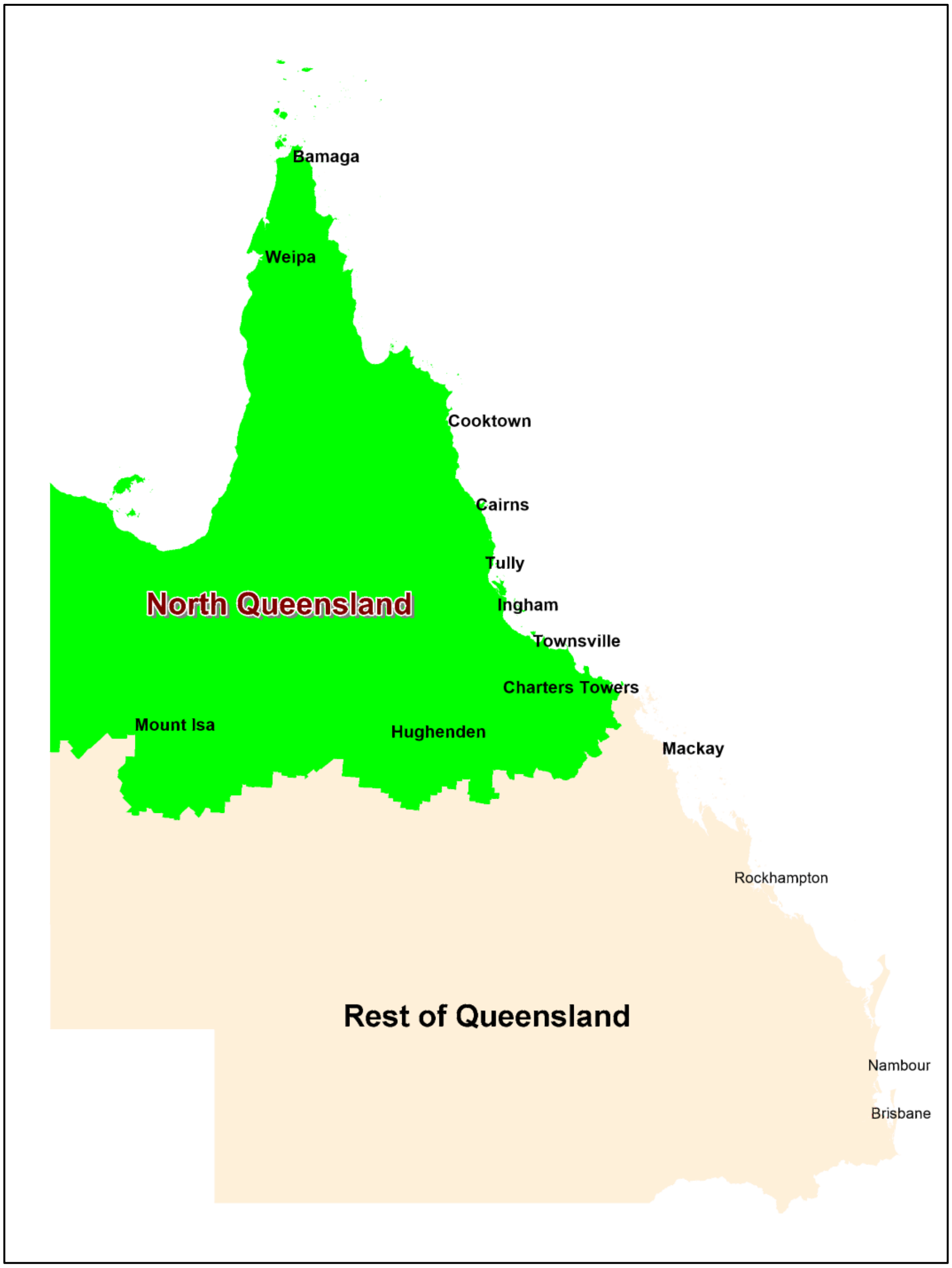

Figure S1.2 Estimated resident population (ERP) of women aged 20-69 years eligible for cervical screening, by Aboriginal and Torres Strait Islander status and Statistical Area Level 2 (SA2), Queensland, Australia, 2013-2017.

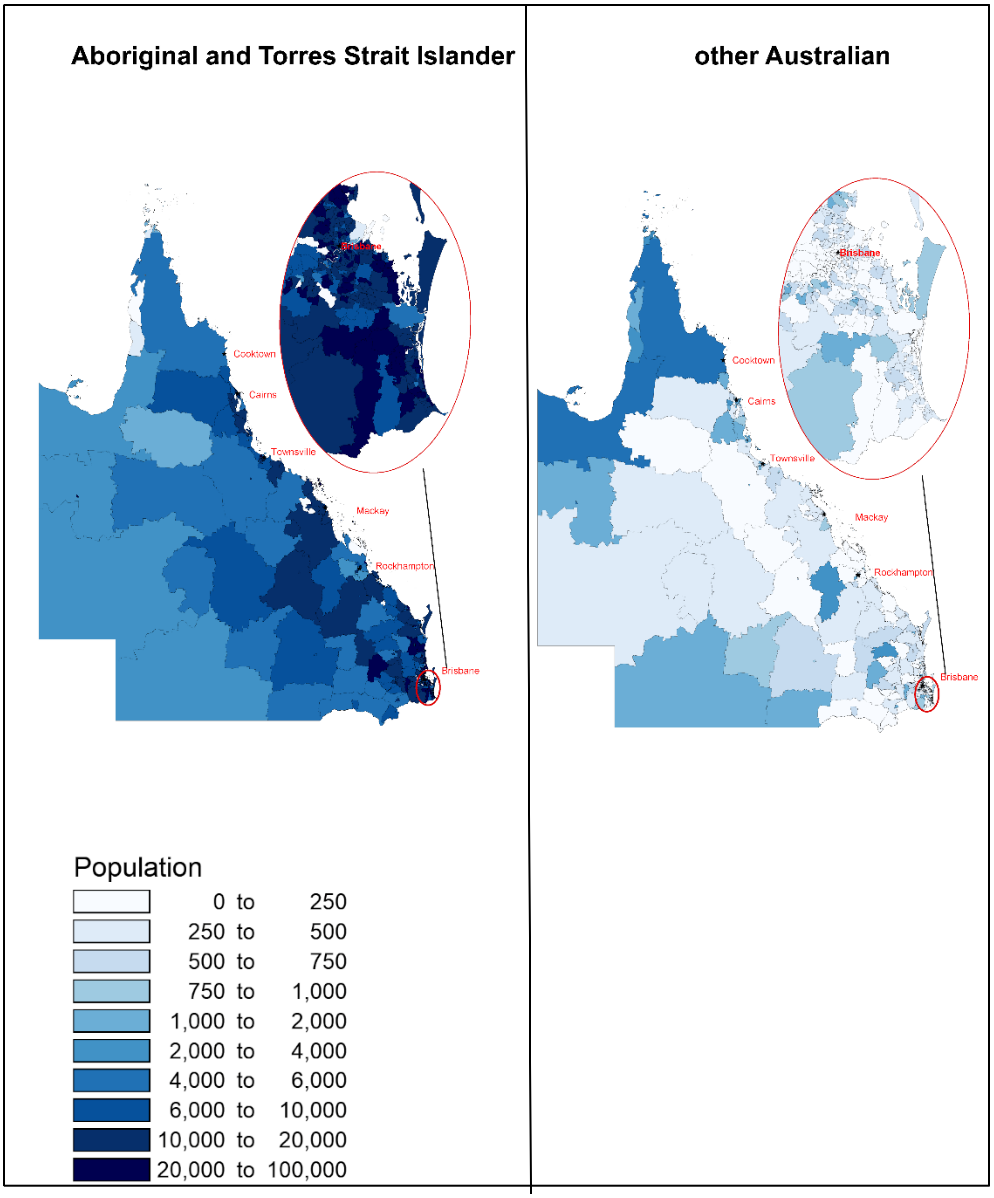

3 **Figure S1.3 Distribution of Aboriginal Community-Controlled Health Organisations (ACCHOs) by estimated resident population (ERP) of Aboriginal and Torres Strait Islander women aged 20-69 years eligible for cervical screening, by Statistical Area Level 2 (SA2), Queensland, Australia, 2013-2017.**

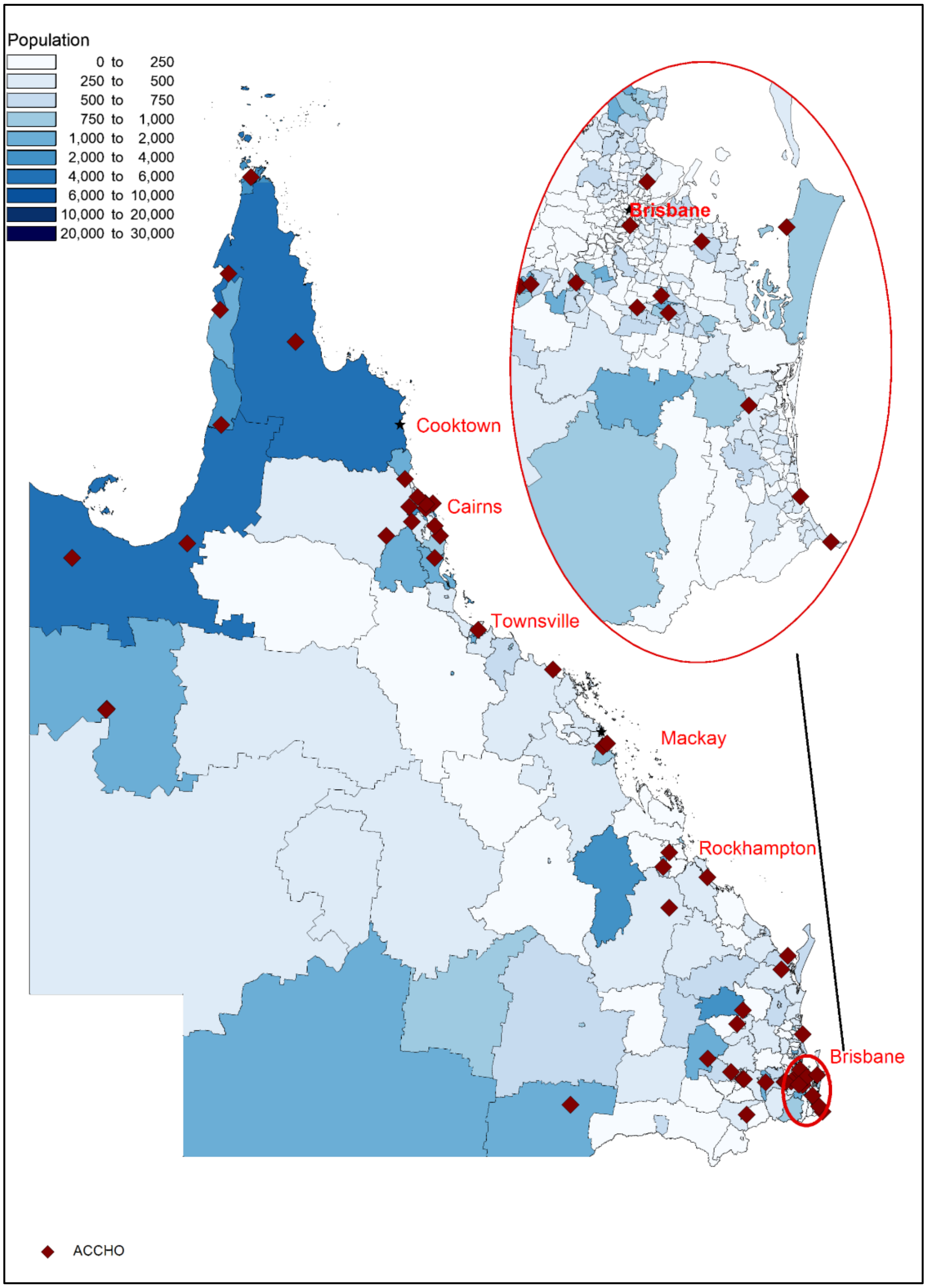

Supplement: Supplementary file 1 [file DataSheet_1.pdf]
