# Supplementary material for: Access to Aboriginal Community-Controlled Primary Health Organizations Can Explain Some of the Higher Pap Test Participation Among Aboriginal and Torres Strait Islander Women in North Queensland, Australia
Source: Front Oncol. 2021 Jul 28;11:725145. doi: 10.3389/fonc.2021.725145 (PMC8355598; doi:10.3389/fonc.2021.725145)
Supplement: Supplementary file 2 [file DataSheet_2.pdf]

Supplementary file 2

1 Number of eligible women and age-standardised five-year Pap test participation rates by Aboriginal and Torres Strait Islander status and region, Queensland, 2013-2017.

	North Queensland				Rest of Queensland			
	Aboriginal and Torres Strait Islander		other Australian		Aboriginal and Torres Strait Islander		other Australian	
**Variable**	N (eligible women)	ASR/100	N (eligible women)	ASR/100 [1,2]	N (eligible women)	ASR/100	N (eligible women)	ASR/100 [1,2]
**Overall**	20,302	57.0 [55.9, 58.1]	142,344	85.0 [84.6, 85.6]	33,029	44.2 [43.5, 45.0]	1,222,677	78.6 [78.4, 8.8]
**Age group (years)**								
20-29	6,642	60.3 [58.4, 62.2]	31,911	85.6 [84.6, 86.6]	11,040	46.5 [45.2, 47.8]	290,662	77.5 [77.2, 77.8]
30-39	4,922	58.5 [56.3, 60.6]	32,321	82.0 [81.1, 83.0]	7,749	41.3 [39.9, 42.7]	279,647	79.3 [78.9, 79.6]
40-49	4,385	54.5 [52.3, 56.7]	31,984	79.9 [78.9, 80.9]	7,255	37.4 [36.0, 38.8]	268,938	75.8 [75.5, 76.2]
50-59	2,915	47.6 [45.2, 50.2]	27,303	74.1 [73.0, 75.1]	4,636	32.9 [31.3, 34.6]	220,563	70.3 [70.0, 70.7]
60-69	1,438	42.9 [39.6, 46.5]	18,825	62.1 [61.0, 63.3]	2,349	26.4 [24.4, 28.6]	162,867	60.0 [59.6, 60.3]
**Aboriginal and Torres Strait Islander female (%)[3]**								
Low (<2.0%)	127	35.0 [25.5, 47.2]	5,920	87.1 [84.7, 89.6]	10,955	31.9 [30.9, 33.0]	731,834	78.3 [78.1, 78.5]
High (≥2.0%)	20,175	54.1 [53.1, 55.2]	136,424	78.0 [77.6, 78.5]	22,074	41.2 [40.3, 42.0]	490,843	67.8 [67.6, 68.1]
**Area-level disadvantage[4]**								
Most advantaged	230	29.2 [21.7, 38.5]	6,759	73.0 [70.9, 75.1]	3,002	30.5 [28.5, 32.7]	267,100	82.5 [82.2, 82.9]
Advantaged	1,217	42.1 [38.4, 46.1]	26,451	83.5 [82.4, 84.6]	4,894	34.1 [32.5, 35.8]	292,129	76.0 [75.7, 76.3]
Middle SES	3,102	48.2 [45.8, 50.8]	34,114	78.6 [77.7, 79.5]	6,876	33.5 [32.1, 34.9]	274,804	72.6 [72.3, 72.9]
Disadvantaged	4,703	55.7 [53.5, 57.9]	41,828	75.9 [75.0, 76.7]	7,722	40.8 [39.4, 42.3]	196,922	71.3 [71.0, 71.7]
Most disadvantaged	11,050	56.9 [55.4, 58.3]	33,192	78.8 [77.8, 79.7]	10,535	43.2 [41.9, 44.5]	191,722	64.4 [64.1, 64.8]
**Remoteness[5]**								
Major cities	0	0	0	0	18,275	37.1 [36.2, 38.0]	896,440	77.4 [77.3, 77.6]
Inner regional	0	0	0	0	10,426	35.9 [34.7, 37.1]	261,152	62.6 [62.3, 62.9]
Outer regional	12,481	51.4 [50.2, 52.7]	131,823	79.7 [79.2, 80.2]	3,323	46.2 [43.9, 48.6]	54,454	75.1 [74.3, 75.8]
Remote	3,155	60.2 [57.3, 63.1]	7,375	64.5 [62.6, 66.3]	397	50.1 [43.2, 57.5]	5,819	70.3 [68.1, 72.5]
Very remote	4,666	57.1 [54.9, 59.6]	3,146	58.2 [55.6, 61.0]	608	53.5 [47.7, 59.7]	4,812	72.3 [70.2, 75.0]
**Closest Pap test provider[6,7]**								
ACCHO	18,247	60.8 [57.4, 64.6]	124,541	78.8 [78.3, 79.3]	25,819	37.5 [36.7, 38.3]	1,005,350	73.8 [73.4, 74.2]
Non-ACCHO	2,055	51.5 [49.3, 53.3]	17,803	75.9 [74.6, 77.2]	7,210	40.5 [39.1, 42.0]	217,327	74.1 [73.7, 74.5]
**Number ACCHO providers[6,8]**								
None	9,142	48.9 [47.5, 50.4]	110,772	78.6 [78.0, 79.1]	26,680	35.5 [34.8, 36.3]	1,103,511	74.4 [74.2, 74.5]
One	2,187	53.8 [50.8, 57.1]	10,725	85.3 [83.5, 87.1]	3,858	44.9 [42.7, 47.1]	79,279	69.5 [68.9, 70.1]
Two to four	4,863	53.2 [50.9, 55.2]	17,774	72.8 [71.6, 74.1]	2,308	55.0 [52.0, 58.1]	37,977	74.9 [74.1, 75.8]
Five or more	4,110	66.8 [64.3, 69.5]	3,073	81.5 [78.3, 84.9]	183	62.7 [51.5, 75.7]	1,910	83.3 [79.2, 87.6]
**Number non-ACCHO providers[6,8]**								
None	2,415	44.5 [40.1, 48.0]	10,771	66.5 [64.9, 68.0]	1,041	26.6 [23.4, 30.0]	33,511	63.1 [62.2, 63.9]
One	1,962	56.5 [52.5, 59.5]	10,901	78.1 [76.4, 79.8]	1,501	31.4 [28.5, 34.4]	68,090	70.4 [69.7, 71.0]

	North Queensland				Rest of Queensland			
	Aboriginal and Torres Strait Islander		other Australian		Aboriginal and Torres Strait Islander		other Australian	
**Variable**	N (eligible women)	ASR/100	N (eligible women)	ASR/100 [1,2]	N (eligible women)	ASR/100	N (eligible women)	ASR/100 [1,2]
Two to four	6,473	51.5 [49.7, 53.4]	49,039	77.1 [76.3, 77.8]	8,162	35.5 [34.2, 36.8]	291,099	71.4 [71.1, 71.7]
Five to nine	5,422	60.4 [58.3, 62.5]	36,764	82.8 [81.9, 83.8]	11,697	35.9 [34.8, 37.0]	462,917	75.0 [74.7, 75.2]
10 or more	4,030	47.6 [45.5, 49.9]	34,869	79.5 [78.5, 80.4]	10,628	44.8 [43.5, 46.1]	367,060	76.8 [76.5, 77.1]
**Travel time closest ACCHO provider**[6,7]								
<30 min	16,660	54.5 [51.7, 56.0]	122,290	78.8 [78.3, 79.3]	25,296	37.2 [36.5, 38.0]	997,621	74.0 [73.9, 74.2]
30 min-1 hour	2,847	52.9 [51.7, 54.0]	11,454	74.7 [70.0, 79.5]	6,196	40.1 [38.5, 41.7]	190,171	73.6 [73.2, 74.0]
1-2 hours	738	50.1 [45.1, 55.5]	7,719	73.9 [72.0, 75.9]	1,214	40.9 [37.3, 44.7]	32,613	77.6 [76.6, 78.5]
2-5 hours	57	45.3 [27.8, 69.6]	881	80.6 [74.7, 86.9]	323	60.0 [51.7, 69.3]	2,272	76.2 [72.6, 80.0]
**Travel time closest non-ACCHO provider**[6,7]								
<30 min	16,946	51.3 [50.2, 52.4]	137,725	78.8 [78.4, 79.3]	31,943	37.8 [37.1, 38.5]	1,206,980	74.0 [73.9, 74.2]
30 min-1 hour	1,859	58.4 [55.8, 61.0]	3,660	64.4 [62.1, 66.3]	990	45.8 [41.7, 50.2]	15,140	75.5 [74.1, 76.9]
1-2 hours	349	42.3 [35.5, 49.5]	41	49.7 [29.1, 81.5]	96	75.3 [58.6, 95.2]	557	86.1 [78.3, 94.4]
2-5 hours	1,148	55.2 [51.8, 57.9]	918	65.3 [45.3, 86.7]	0	0	0	0

ASR Age-standardised participation rate, ACCHO Aboriginal Community-Controlled Health Organisation, CI Confidence Interval,

1. Pap test participation rate is the number of women screened at least once in each specified time-period and age group divided by the averaged estimated eligible resident female population for the same time-period and age group, age-standardised to the 2001 Australian standard population. Women who have had a hysterectomy are excluded from the eligible population.
2. Periods covered apply to calendar years.
3. Based on 2016 Census
4. Area-level disadvantage was defined by the 2016 SEIFA Index of Relative Socioeconomic Advantage and Disadvantage
5. Remote areas were defined by the Remoteness Areas 2016 classification.
6. Provider refers to provider of a Pap test and are based on medical centres or general practitioner practices. One centre or practice may have multiple health professionals who provide Pap tests.
7. Based on travel distance from 2016 (SA2) at screening to geocoded street address of a Pap test provider
8. Number providers by 2016 Australian Statistical Geography Statistical Area Level 2 (SA2) for a woman at screening
